# Quantification of aortic valve regurgitation by phase-contrast magnetic resonance in patients with bicuspid aortic valve: where to measure the flow?

**DOI:** 10.1186/1532-429X-15-S1-O37

**Published:** 2013-01-30

**Authors:** Stefano Muzzarelli, Pierre Monney, Kieran O'Brien, Francesco Faletra, Tiziano Moccetti, Pierre Vogt, Juerg Schwitter

**Affiliations:** 1 Cardiology, Fondazione Cardiocentro Ticino, Lugano, Switzerland; 2 Cardiology, University Hospital Lausanne, Lausanne, Switzerland; 3 CIBM, University of Geneva, Geneva, Switzerland

## Background

Phase-contrast magnetic resonance (PC-MR) is used to quantify the aortic regurgitation (AR) by measuring the forward and backward flow in the ascending aorta (AAo), thereby quantifying the regurgitant fraction. Patients with bicuspid aortic valve (BAV) have an eccentric systolic aortic flow jet, causing an abnormal flow in the AAo. Therefore, we hypothesized that the flow measurement in the AAo in BAV patients leads to an underestimation of the forward aortic flow and a consequent overestimation of the AR.

## Methods

Flow measurement by PC-MR was performed in 22 BAV patients and 20 controls at the following positions: 1) left ventricular outflow tract (LVOT), 2) aortic valve orifice (AV), and 3) AAo. The forward flow measured in these locations was compared with the left ventricular stroke volume (LVSV) in BAV patients and controls. Finally, the severity of the AR was quantified. Intravoxel dephasing was estimated by the ratio of the mean signal intensity on magnitude images across the vessel between systole and diastole.

## Results

**Figure 1:**
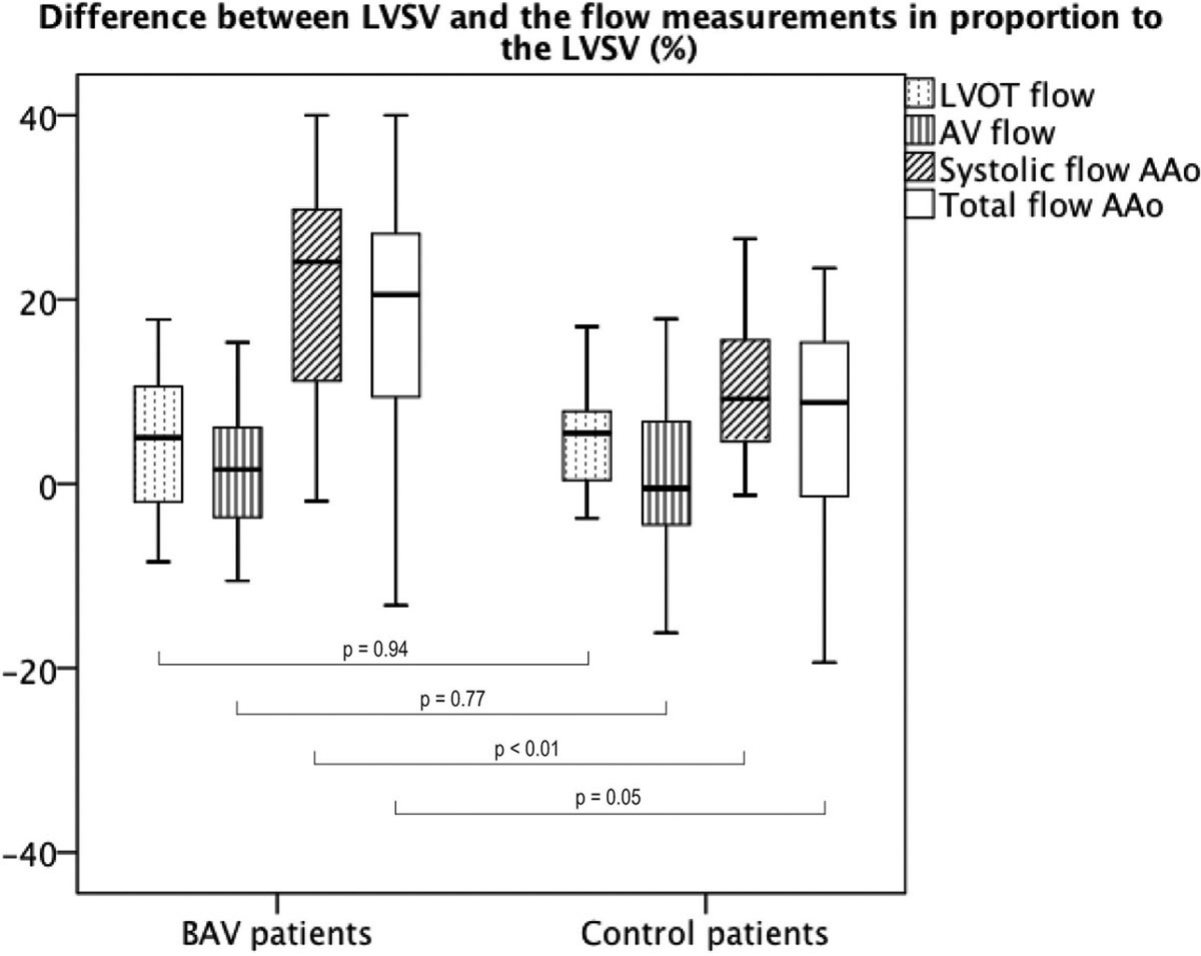
Box-plots showing the relative difference (%) between the left ventricular stroke volume and the flow measurement performed in the different locations [(left ventricular stroke volume - forward flow) / left ventricular stroke volume] x 100. Notably, there was a mild difference between the left ventricular stroke volume and forward flow in the left ventricular outflow tract and the aortic valve orifice, which did not differ between normal BAV patients and controls. Conversely, in the ascending aorta there was a slight underestimation in forward flow among controls, which was much more pronounced and significant in BAV patients, especially if the systolic component only of the forward flow was considered. LVOT, left ventricular outflow tract; AV, aortic valve; AAo, ascending aorta.

The correlation between the LVSV and the forward flow in the LVOT, the AV and the AAo was good in both BAV patients (r= 0.97/0.96/0.86; p < 0.01 for all) and controls (r= 0.96/0.93/0.87; p < 0.01 for all). However, in relation with the LVSV, the forward flow in the AAo was mildly underestimated in controls and much more in BAV patients [median (interquartile range): 9% (-1%/16%) vs. 17% (7%/27%); p = 0.05]. This was not the case in the LVOT [5% (0%/8%) vs. 5% (-3%/11%); p = 0.94] and the AV [0% (-5%/8%) vs. 1% (-4%/6%); p = 0.77], where the differences were mild and did not differ between groups (figure 1). By applying cut-off values of regurgitant fraction in the AAo, the severity of the AR was overestimated in 6 out of 16 BAV patients (38%) with AR, as compared with the flow quantification in the LVOT or the AV. In the AAo the relative mean SI was lower in the BAV patients compared to controls [1.34 +/- 0.15 vs. 1.58 +/- 0.26; p < 0.01], suggesting more intravoxel dephasing in BAV patients.

## Conclusions

Flow measurement in the AAo by PC-MR leads to a significant underestimation of the forward aortic flow and a consequent overestimation of the AR in BAV patients. Flow measurement in the LVOT or the AV better correlates with the LVSV, indicating an alternative means for quantifying the aortic regurgitation in BAV patients.

## Funding

None.

